# Discrimination and reliability: equal partners? Understanding the role of discriminative instruments in HRQoL research: can Ferguson's Delta help? A response

**DOI:** 10.1186/1477-7525-6-83

**Published:** 2008-10-16

**Authors:** Matthew Hankins

**Affiliations:** 1 King's College London, Department of Psychology (at Guy's), Institute of Psychiatry, London, UK; 2 Department of Primary Care & Public Health, Brighton & Sussex Medical School, Brighton, UK; 3 Brighton & Sussex University Hospitals NHS Trust, Royal Sussex County Hospital, Brighton, UK

## Abstract

A response to Norman GR 'Discrimination and reliability: equal partners?' and Wyrwich KW 'Understanding the role of discriminative instruments in HRQoL research: can Ferguson's Delta help?'

## Response

I would like to thank Norman and Wyrwich for their close reading of my article [1], and also the editors for inviting this debate. It is a welcome opportunity to clarify some points and expand upon others.

I should like to begin by re-stating what coefficient Delta *is *and what it *is not*. Delta is the ratio of observed discriminations made to the maximum possible number; it ranges from 0 (no discriminations at all are made) to 1 (all possible discriminations are made for a given sample size and scale range). *Discriminations *means between-persons differences, which is to say, two people are discriminated if they score differently on the instrument, and not discriminated if they score the same. This definition is in keeping with Kirshner's & Guyatt's [2] definition of a discriminative instrument and Norman's second dictionary definition. Delta is not a substitute for a reliability coefficient, since reliability is an index of measurement error, nor is it a substitute for a validation correlation coefficient, since this refers to the extent to which the instrument measures the correct construct. In fact, unless an instrument has been shown to be valid and reliable, there is little to be gained in assessing its discrimination. What I have argued, however, is that *validity and reliability alone fail to establish that a discriminative instrument achieves its purpose of discriminating between individuals*.

Hence the examples in the article take validity and reliability as givens; it is assumed that anyone interested in the discrimination of an instrument has already established that the instrument is reliable and valid, by whatever means they find acceptable. The issues of which reliability coefficient is used, or how exactly validity is established, are irrelevant. But Norman and Wyrwich make much of the examples and their (apparent) shortcomings in this regard. This seems to me to miss the point: the examples are to illustrate the utility of Delta as an *additional *index of an instrument's measurement properties. They are not complete examples of the development of a HRQoL instrument (Wyrwich's chief complaint), nor do they suggest that Delta should replace the reliability coefficient (Norman's main objection). In Example 1 the ICC is not intended to be a reliability coefficient, as suggested by both authors, but simply a measure of agreement between the two scales (Norman states, 'all reliability coefficients are ICCs', which it true, but not all ICCs are reliability coefficients).

Norman also suggests that the discrimination of an instrument is *already *indexed by the reliability coefficient, citing his own textbook [3]: 'the reliability coefficient reflects the extent to which a measurement instrument can differentiate among individuals, *since the magnitude of the coefficient is directly related to the variability between subjects"*, and later,' reliability is a measure of the extent to which people differ, expressed as a number between 0 and 1'. This is simply incorrect. The magnitude of the reliability coefficient tells us *nothing *about the variability between subjects, and *nothing *about the extent to which people differ: indeed, this is the whole point of my argument. Norman correctly states that the reliability coefficient reflects the 'proportion of the variance in the observations that relate to real differences among subjects'. Proportions 'lose' the quantity of interest, since it appears in both the numerator and denominator. A reliability coefficient of 0.8, for example, tells us that 80% of the observed variance is due to true score variance, but it does not tell us what either variance *is*. Two scales might have reliability coefficients of 0.8, but wildly different variances. Hence, as argued, reliability coefficients do not serve the purpose of quantifying the degree of discrimination offered by an instrument. They do, however, establish the consistency with which discriminations are made.

Both Norman and Wyrwich raise the interesting issue of what constitutes a *meaningful *discrimination, and whether Delta is an index of such discriminations. This happens to be a valid point, but not as argued here. Norman argues it in terms of measurement error (how can we be sure that discriminations are 'real' if some of them are due to measurement error?) and Wyrwich from the perspective of interpretability (what size of discrimination should be considered important?). Implicit in this argument is that a measure of discrimination *would *be useful if the discriminations observed were meaningful. Recall that the value of Delta is derived from ordinal comparisons of persons that classify them as either *the same *or *different*. Therefore, if a researcher doubts that an instrument can be trusted to make this most basic distinction, then the instrument *should not be used*. It makes no sense to declare that an instrument has 'acceptable' reliability and interpretability, but then argue that it cannot be trusted to rank order people in a meaningful way. This would invalidate *any *statistical treatment of data that failed to take measurement error into account. It does not constitute an argument against the use of Delta.

It is possible, however, to incorporate these elements into the computation of a coefficient of discrimination if required. As Thurlow [4] pointed out, the only discriminations worth considering are *valid discriminations*. Ferguson's Delta [5] is computed on the assumption that the measurement is valid and reliable to the degree that the instrument produces a valid rank ordering of people (number of discriminations observed/total number possible). If this is not the case, then the numerator should be adjusted to take into account only meaningful differences, however defined.

For example, if the reliability coefficient suggests that differences in the observed score should be greater than 3 points to allow for measurement error, then a '*discrimination' *becomes '*any between-person difference of greater than 3 points'*. Similarly, if the minimum important difference is considered to be 5 points, then a discrimination is defined as any between-persons difference > = 5 points. Delta then indexes the degree to which the instrument makes valid discriminations.

Wyrwich suggests that the results of another study [6], in which the dichotomous scoring method of the GHQ-12 was found to be less discriminating than the Likert scoring method, were 'well expected'. This again suggests that an index of discrimination serves a useful purpose as an empirical test of assumptions such Wyrwich's: 'Likert response items (if chosen correctly) are more discriminating between individuals than dichotomous items'. In fact, this is not always true: for example, the variant dichotomous scoring method for the GHQ-12 [7] can result in *greater *discrimination than the Likert scoring method [8].

I suspect that Norman, Wyrwich and I agree on the fundamentals: discriminative HRQoL instruments should be validated and of sufficient reliability for the task at hand; they should provide interpretable data; and thus any discriminations made should be 'real'. My argument is that the degree to which a discriminative instrument actually discriminates between people should be quantified by a separate index, Delta. It remains to be seen how these elements, particularly reliability and discrimination, interact.

In closing I should directly answer the questions posed by Norman and Wyrwich in the titles of their pieces. Norman asks 'Discrimination and reliability: Equal partners?' to which the answer is *no*; reliability trumps discrimination, for reasons explained above. Wyrwich asks 'Understanding the Role of Discriminative Instruments in HRQoL Research: Can Ferguson's Delta Help?' to which the answer is a definitive *yes*, subject to the constraints previously discussed.

Finally, I have a question for them. You are faced with the choice of two discriminative HRQoL instruments, A and B. Both are reliable enough for your purposes; they are also equally valid. In all other respects they meet your requirements equally well. Delta for instrument A is 0.95, and for instrument B it is 0.30.

Which would you choose?

## List of abbreviations

HRQoL: Health related quality of life; GHQ-12: General health questionnaire (12 item version).

## Competing interests

The author declares that they have no competing interests.

## Author information

MH is a Senior Research Fellow in the Division of Primary Care & Public Health, Brighton & Sussex Medical School, United Kingdom.
